# Similarity Score for the Identification of Active Sites in Patients With Atrial Fibrillation

**DOI:** 10.3389/fphys.2021.767190

**Published:** 2022-01-20

**Authors:** Vasanth Ravikumar, Sanket Thakare, Xiangzhen Kong, Henri Roukoz, Elena G. Tolkacheva

**Affiliations:** ^1^Department of Electrical and Computer Engineering, University of Minnesota, Minneapolis, MN, United States; ^2^Department of Biomedical Engineering, University of Minnesota, Minneapolis, MN, United States; ^3^Division of Cardiology, Department of Medicine, University of Minnesota, Minneapolis, MN, United States

**Keywords:** atrial fibrillation, ablation, signal processing, earth mover’s distance (EMD), similarity score

## Abstract

**Background:**

Atrial fibrillation (AF) is the most common cardiac arrhythmia and precursor to other cardiac diseases. Catheter ablation is associated with limited success rates in patients with persistent AF. Currently, existing mapping systems fail to identify critical target sites for ablation. Recently, we proposed and validated several individual techniques, such as dominant frequency (DF), multiscale frequency (MSF), kurtosis (Kt), and multiscale entropy (MSE), to identify active sites of arrhythmias using simulated intracardiac electrograms (iEGMs). However, the individual performances of these techniques to identify arrhythmogenic substrates are not reliable.

**Objective:**

This study aimed to develop a similarity score using various iEGM analysis techniques to more accurately identify the spatial location of active sites of arrhythmia in patients with AF.

**Methods:**

Clinical bipolar iEGMs were obtained from patients with AF who underwent either successful (*m* = 4) or unsuccessful (*m* = 4) catheter ablation. A similarity score (0–3) was developed via the earth mover’s distance (EMD) approach based on a combination of DF, MSF, MSE, and Kt techniques.

**Results:**

Individual techniques successfully discriminated between successful and unsuccessful AF ablation patients but were not reliable in identifying active spatial sites of AF. However, the proposed similarity score was able to pinpoint the spatial sites with high values (active AF sites) that were observed only in patients with unsuccessful AF termination, suggesting that these active sites were missed during the ablation procedure.

**Conclusion:**

Arrhythmogenic substrates with abnormal electrical activity are identified in patients with unsuccessful AF termination after catheter ablation, suggesting clinical efficacy of similarity score.

## Introduction

According to an estimate in 2014, 2.7–6.1 million people in the United States are afflicted by atrial fibrillation (AF). AF is the most common type of supraventricular arrhythmia in the United States and is associated with an increased risk of stroke (Go et al., 2001; Miyasaka et al., 2006). AF is characterized by the chaotic electrical activity in the atria (Munger et al., 2014). Furthermore, the prevalence of AF has been projected to increase to nearly 12.1 million people by 2050 (Miyasaka et al., 2006; Colilla et al., 2013). These reasons make AF a significant public health concern and underpin the need to develop more efficient treatment techniques.

Compared with antiarrhythmic drug therapy, catheter ablation improves AF symptoms and quality of life and reduces AF recurrence (Wazni et al., 2005; Jaïs et al., 2008; Wilber et al., 2010). Pulmonary vein (PV) isolation, the most commonly used catheter ablation approach, alone can be used as a strategy for catheter ablation in patients with paroxysmal AF (Sawhney et al., 2009; Ouyang et al., 2010; Chao et al., 2012). However, in the case of persistent and long-standing AF, electrical initiation of AF may arise outside the PV regions, which play an essential role in arrhythmogenesis and maintenance of AF (Elayi et al., 2010). Hence, it is important to identify AF ablation targets outside the PV regions for the improved success of catheter-based ablation therapy.

Several approaches, such as local activation maps, phase maps, and dominant frequency (DF) maps (Pandit and Jalife, 2013), are commonly used to identify ablation target sites. In our laboratory, we developed several novel techniques to identify active sites of AF, especially outside the PV regions, both in the frequency-domain [multiscale frequency (MSF) (Arunachalam et al., 2016b)] and time-domain [multiscale entropy (MSE) (Arunachalam et al., 2018) and kurtosis (Kt) (Arunachalam et al., 2016a)]. All novel techniques have been validated using optical mapping studies of *ex vivo* rabbit hearts and have been established to accurately identify the active sites or arrhythmias, such as pivot point of rotors in experiments where rotors have been visually observed (Annoni et al., 2018). These techniques were shown to be accurate under some clinical limitations, such as reduced signal time duration and decreased spatial resolution. It was also demonstrated that the novel techniques could differentiate between the intracardiac electrograms (iEGMs) recorded from patients with successful and unsuccessful AF ablation (Ravikumar et al., 2021). However, the use of individual approaches fails to robustly identify electrically active spatial sites for ablation (Ravikumar et al., 2021), and therefore, efforts on developing combinatory approaches to identify the active AF sites are needed.

In this study, we aimed to develop a similarity score by combining the various iEGM analysis approaches (DF, MSF, MSE, and Kt) based on an earth mover’s distance (EMD) method. We further demonstrated that this similarity score can identify active spatial sites of AF in patients with unsuccessful AF termination, while no active sites of AF were present in patients with successful AF termination.

## Materials and Methods

### Patient Population and Clinical Intracardiac Electrograms Data

All the patients (*m* = 8) had persistent AF and underwent PV isolation (PVI) during ablation therapy. Successful PVI was defined as the elimination or dissociation of all PV potentials recorded. Complete isolation of PVs was achieved in all cases. In patients whose rhythms were reorganized to atrial tachycardia (AT) or atrial flutter, mapping and ablation of the tachycardia were performed to restore sinus rhythm. In this study, successful AF termination was defined as the acute termination of the AF during PVI or after conversion into organized AT or atrial flutter before conversion into sinus rhythm, either spontaneous or with ablating the residual AT. In patients with unsuccessful AF termination, a further cardioversion procedure was performed to terminate AF, and therefore, unsuccessful AF termination was defined in patients who underwent cardioversion to restore sinus rhythm. The baseline characteristics of all patients are listed in Supplementary Table 1.

Clinically recorded bipolar iEGMs (BiEGMs) were obtained from the left atrium (LA) from *m* = 8 patients, with prior approval under the Institutional Review Board (IRB: STUDY00003128) of the University of Minnesota. All experiments were performed as per relevant guidelines and regulations, and the consent of the patient was obtained before obtaining the data. Simultaneous iEGM collection was carried out using the CARTO (Biosense Webster, Irvine, CA, United States) system, which has a sensor position accuracy of 0.8 mm and 5°. BiEGMs were recorded with a sample rate of 977 Hz and a duration of 5–15 s at different spatial sites (*N* = 16–24) in each patient, using high-resolution PentaRay catheters (Biosense Webster, Irvine, CA, United States). A spatial site was defined as the unique placement of the PentaRay catheter in the atria so that 10 BiEGMs were recorded and individually analyzed from each spatial site. Furthermore, the distribution of the metrics obtained from 10 BiEGMs in each site for the various approaches was compared using the EMD. Notably, 160–240 BiEGMs were recorded before the PVI ablation procedure.

Three independent reviews were performed to identify the noisy, low amplitude, and contact loss signals. BiEGMs with extremely low amplitude, high noise corruption, loss of contact, and low signal-to-noise ratio were removed, and only the good signals were used for the retrospective analysis. BiEGMs were then filtered with a third-order IIR Butterworth band-pass filter 3–15 Hz to maintain the frequency components of the recorded signals in the physiological range.

### Similarity Score Based on Earth Mover’s Distance Method

To develop a similarity score, four different approaches that were previously used in the literature for the BiEGM analysis, namely, MSF (Arunachalam et al., 2016b), MSE (Arunachalam et al., 2018), DF (Sanders et al., 2005), and Kt (Arunachalam et al., 2016a), were used. More details of these techniques are provided in Supplementary Table 2. We have chosen these techniques for the initial development of similarity scores since they represent different characteristics of the BiEGM signals such as frequency, information content, and amplitude-based statistics. In this study, we have not examined the contribution of each individual approach to the outcomes of similarity score and therefore assigned equal weights to each approach. Furthermore, the similarity score can be optimized based on various weighted contributions from a different signal processing approaches.

The similarity score is based on the EMD approach (Rubner et al., 2000) and compares the performance of pairs of different individual techniques at various spatial sites in the atria of the patient. A high similarity score indicates that the individual techniques identified the same spatial site as the active site of AF.

The EMD is used to evaluate the similarity between two multidimensional distributions and is based on a solution to the well-known transportation problem (Rubner et al., 2000). First, let ***P*** = {*p*_*n*,1_, *p*_*n*,2_, …, *p*_*n,J*_} and ***Q*** = {*q*_*n*,1_, q_*n*,2_, …, *q*_*n,J*_} be the distributions of values from two different approaches for *J* = 10 BiEGMs, respectively, recorded at each site *n*. Second, we defined the Euclidean distance-vector, containing the distance between ***P*** and ***Q*** elements. Then, at each spatial site *n*, we calculated *EMD*_*n*_(**P**, **Q**) as follows:


(1) $$EMD_{n}\left(P,Q\right)=\frac{\sum_{i=1}^{J}\sum_{j=1}^{J}f_{ij}d_{ij}}{\sum_{i=1}^{J}\sum_{j=1}^{J}f_{ij}}$$

where *n* = [1, 2, …, *N*] is spatial sites, *f*_*ij*_ is the flow rate between elements pni and qnj, and *d*_*ij*_ is the distance between elements pni and qnj. For example, using our recorded data from patient 1, we have *N = 18* sites. Therefore, we obtained 18 values of *EMD*_*n*_(**P**, **Q**). The value of EMD = 0.2 was considered as a threshold for similarity, i.e., if two approaches have EMD < 0.2 (Ravikumar et al., 2018), they are similar, i.e., they have a strong correlation. The threshold was selected based on the minimal distances between the distributions. Furthermore, this threshold indicates that matches have a similarity greater than 0.8 on a scale of 0–1 for the minimal distances between the distributions to be satisfied.

The EMD values were calculated for all approaches, and the following pairs were calculated for all the patients at each spatial site: (1) DF vs. MSF, (2) DF vs. MSE, (3) MSF vs. MSE, (4) DF vs. Kt, (5) MSF vs. Kt, and (6) MSE vs. Kt. Then, at each spatial site, the number of pairs satisfying the threshold requirements for the EMD similarity index (EMD < 0.2) was computed, ranging from 0 (no pair is above the threshold) to 6 (all pairs are above the threshold). Finally, a *similarity score* of 0–3 was assigned to each spatial site in every patient reflecting the number of pairs above the threshold: 0 (no pairs), 1 (1–2 pairs), 2 (3–4 pairs), and 3 (5–6 pairs). A high *similarity score* of 3 indicates that all four methods identify this specific spatial location as a potential site for AF abnormal activity and subsequent ablation.

### Three-Dimensional Reconstruction Using VIEgram

A custom VIEgram software (Thakare et al., 2020) was used to demonstrate the three-dimensional (3D) visualization of individual approaches using BiEGMs collected from patients with AF. A custom-built Python script was used for data extraction, while data processing and 3D mapping were performed using custom-built MATLAB (MathWorks, Natick, MA, United States) scripts. The 3D images were constructed by superimposing the individual measures on a 3D mesh of the LA, and the individual approaches were correlated using the EMD approach.

### Statistical Analysis

All the statistical analyses were performed using the MATLAB (MathWorks, Natick, MA, United States) software. A statistical Wilcoxon rank-sum test was performed for significance testing to determine whether the individual techniques can differentiate between the iEGMs recorded from patients with successful and unsuccessful AF termination, with *p* < 0.05 being considered as statistically significant. For categorical variables expressed as number and percentage, a Fisher’s exact test was performed.

## Results

**FIGURE 1:**
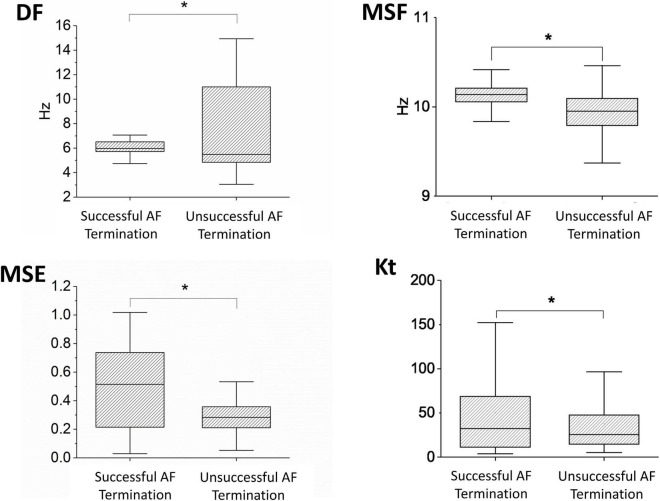
Performance of the different approaches, such as dominant frequency (DF), multiscale frequency (MSF), multiscale entropy (MSE), and kurtosis (Kt), that were applied to the bipolar intracardiac electrogram (BiEGM) analysis from patients with successful (*m* = 4) and unsuccessful (*m* = 4) atrial fibrillation (AF) termination. Asterisk indicates statistical significance with *p* < 0.05.

First, we aimed to demonstrate whether individual techniques (MSF, MSE, DF, and Kt) can discriminate between patients with successful and unsuccessful AF termination when applied to BiEGMs. In Figure 1, the box plots of the MSF, MSE, DF, and Kt values are shown for all patients with successful (*m* = 4) and unsuccessful (*m* = 4) AF termination. Notably, all the methods show statistical significance (*p* < 0.05) between the two groups of patients, suggesting the successful discrimination based on all BiEGMs.

**FIGURE 2:**
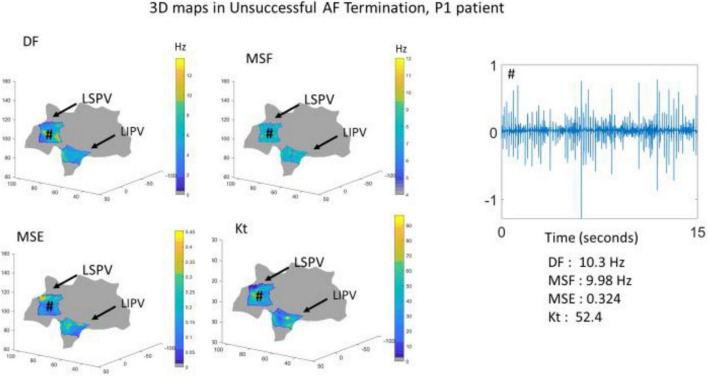
The three-dimensional (3D) visualization of the individual approaches (DF, MSF, MSE, and Kt) generated using VIEgram software for a specific unsuccessful AF termination patient P1. Sample of BiEGM recorded from the left superior pulmonary vein (LSPV) (#) is shown along with individual DF, MSF, MSE, and Kt values.

**FIGURE 3:**
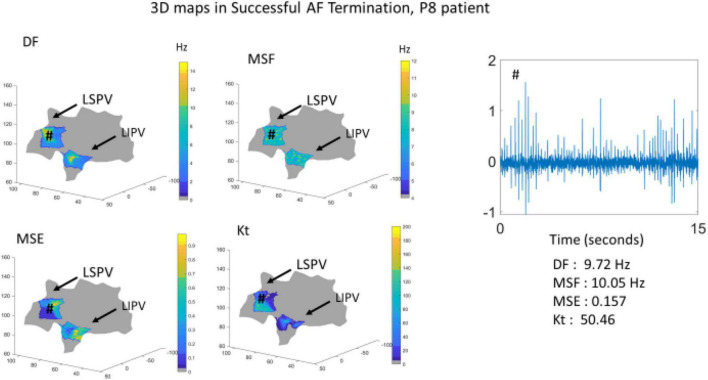
The 3D visualization of the individual approaches (DF, MSF, MSE, and Kt) generated using VIEgram software for a specific successful AF termination patient P8. Sample of BiEGM recorded from LSPV (#) is shown along with individual DF, MSF, MSE, and Kt values.

To further demonstrate the performance of the BiEGM analysis approaches in identifying active sites of AF, the 3D visualization of individual approaches was performed using the custom-made VIEgram software. Figures 2, 3 show the representative examples of 3D maps of DF, MSF, MSE, and Kt obtained via VIEgram software for patients with unsuccessful and successful AF termination, respectively. The atria are visualized to show the left superior PV (LSPV) and the left inferior PV (LIPV). The corresponding example of a BiEGM signal from a single electrode (#) is also shown along with the individual DF, MSF, MSE, and Kt values from this recording. Notably, the visual inspection of spatial similarity between all four approaches does not suggest any correlation between the techniques both in a patient with unsuccessful and successful AF termination. Therefore, individual approaches alone cannot correctly identify the active sites of AF that should only be present in patients with unsuccessful AF termination.

**FIGURE 4:**
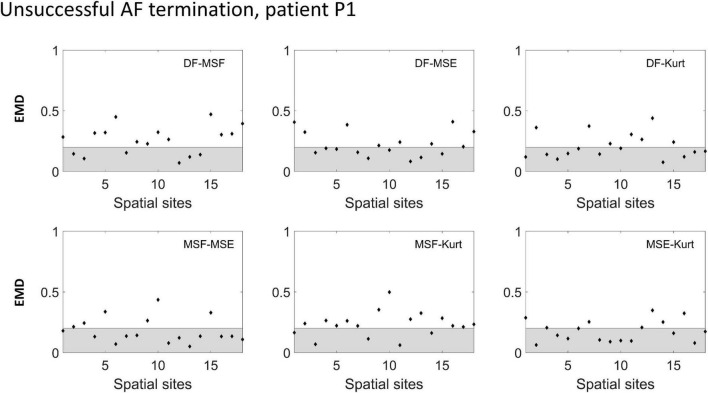
Earth mover’s distance (EMD) values calculated for the different pairs of approaches (DF, MSF, MSE, and Kt) in an unsuccessful AF termination patient P1. The gray area indicates the region where the correlation between the pairs is high (EMD < 0.2).

**FIGURE 5:**
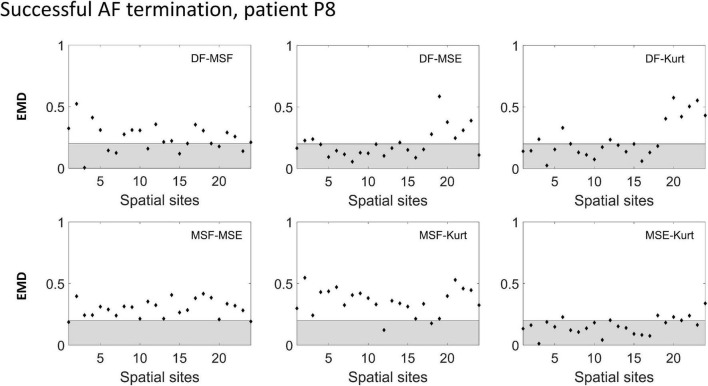
Earth mover’s distance (EMD) values calculated for the different pairs of approaches (DF, MSF, MSE, and Kt) in a successful AF termination patient P8. The gray area indicates the region where the correlation between the pairs is high (EMD < 0.2).

To improve the performance of the various BiEGM analysis techniques for the identification of active AF sites, a similarity score was developed based on EMD to quantify the correlation between various pairs of the techniques at each spatial site. In Figures 4, 5, the representative examples of the EMD values calculated for different pairs of approaches at different spatial sites are shown for patients with unsuccessful and successful AF termination, respectively. The gray shaded area indicates the regions with EMD < 0.2, where all pairs are strongly correlated, and therefore, the similarity score is high. As indicated in Figures 4, 5, different techniques have a different degree of correlation with each other, and the degree of correlation is also different depending on the spatial site. We hypothesized that the spatial sites in which at least 5 pairs of the techniques are correlated, i.e., with a high similarity score of 3, might be associated with active AF sites.

The correlation data from Figures 4, 5 are summarized in Table 1 (patient P1 with unsuccessful AF termination) and Table 2 (patient P8 with successful AF termination), respectively, where the EMD values below (orange) and above (gray) the threshold (EMD = 0.2) are shown for all the spatial sites. Notably, in patient P1 with unsuccessful AF termination, there are three spatial sites (# 3, 8, and 14, red color) in which the similarity score is high (=3). Such a high similarity score indicates that at least 5 pairs of different approaches are correlated in these sites, suggesting the presence of active AF drivers, which most probably were not properly targeted during the AF ablation procedure. However, the absence of such red sites with a high similarity score in a patient with successful AF termination (Table 2) was noted, indicating that all active sites were appropriately targeted during the AF ablation procedure. Similar tables for all remaining patients are present in Supplementary Tables 3–8.

**TABLE 1:**
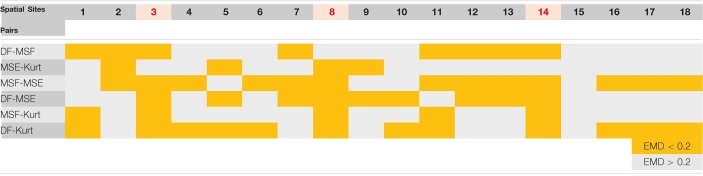
Earth mover’s distance (EMD) correlation between different pairs of approaches for various spatial sites of a representative patient P1 with unsuccessful atrial fibrillation (AF) termination (from Figures 1, 3).

*High/low correlation with respect to the threshold value of EMD = 0.2 is indicated by the orange/gray color. Red spatial sites indicate active AF sites with a similarity score >3, indicating that at least 5 pairs are highly correlated.*

**TABLE 2:**
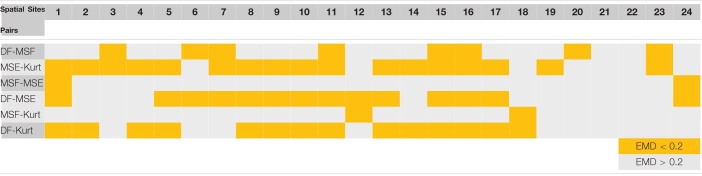
Earth mover’s distance correlation between different pairs of approaches for various spatial sites of a representative patient P8 with successful AF termination (from Figures 2, 4).

*High/low correlation with respect to the threshold value of EMD = 0.2 is indicated by the orange/gray color. Red spatial sites indicate active AF sites with a similarity score >3, indicating that at least 5 pairs are highly correlated.*

**FIGURE 6:**
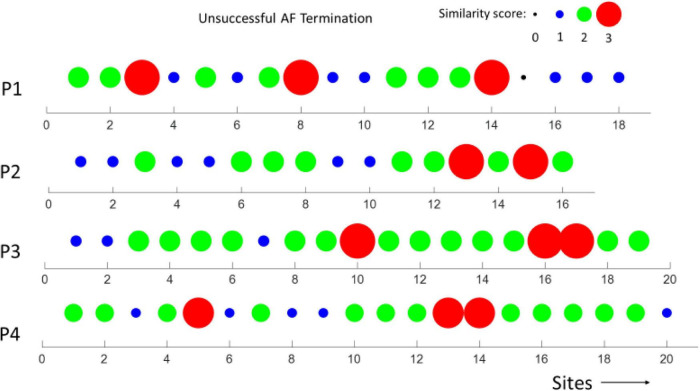
Similarity scores calculated at different spatial sites in *m* = 4 patients with unsuccessful AF termination. Notably, the presence of several spatial sites (potential active AF sites) with a high similarity score (3, red) in all the patients.

**FIGURE 7:**
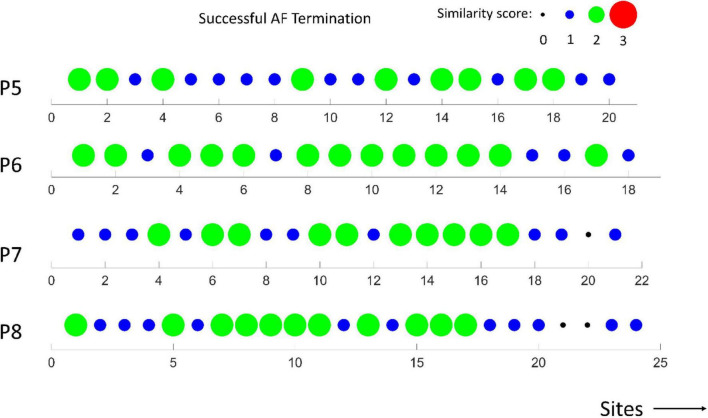
Similarity scores calculated at different spatial sites in *m* = 4 patients with successful AF termination. Notably, the absence of active AF sites with a high similarity score (3).

In Figures 6, 7, the similarity scores for different spatial sites are shown for all patients with unsuccessful (*m* = 4) and successful (*m* = 4) AF termination, respectively. Notably, the spatial sites with high similarity scores (=3, red) are only identified in patients with unsuccessful AF termination, suggesting that these sites can be the active AF sites and potential AF ablation targets. The sites were distributed among the different regions of the heart, namely, LA appendage, lateral wall, roof wall, posterior wall, inferior wall, septal wall, LSPV, and LIPV.

## Discussion

Some recent studies indicated that a PV isolation alone could be a strategy for catheter ablation in patients with paroxysmal AF, while recurrence is not fully prevented (Sawhney et al., 2009; Ouyang et al., 2010; Chao et al., 2012). However, other studies suggested the need for mapping-specific ablation therapy in patients with persistent AF when active AF sites are outside of the PV regions (Barbhayia et al., 2015; Seitz et al., 2017). For instance, it has been shown that EGM dispersion occurs in the vicinity of AF drivers, and this dispersion is present both near and outside the PV regions. Therefore, it is important to develop new techniques for identifying AF substrates outside the vicinity of the PV regions and potentially improving the success rates of the catheter ablation procedure. Several studies demonstrated the successful identification of AF driver by the phase mapping of simultaneous recordings using a basket catheter (Narayan et al., 2012) and a noninvasive array of body surface electrodes (Haissaguerre et al., 2013). It was also demonstrated in the study by Roney et al. (2017) that spatial resolution is essential in identifying the AF reentrant activity. But the reported success rates using these guided strategies for patients with persistent AF remained inconclusive and ranged from 19 to 77% (Narayan et al., 2014; Miller et al., 2017; Mohanty et al., 2018).

Recently, our laboratory developed and validated the novel iEGM analysis techniques, such as MSF (Arunachalam et al., 2016b), MSE (Arunachalam et al., 2018), and Kt (Arunachalam et al., 2016a), to identify the sites with abnormal AF activity that can be a potential target for AF ablation. Furthermore, we demonstrated the feasibility of these techniques in identifying the active sites of arrhythmia under clinical limitations, with acceptable levels of specificities and sensitivities using numerically simulated iEGMs (Ravikumar et al., 2021). Multiple other studies have analyzed other signal processing-based ablation techniques (Lin et al., 2010; Okumura et al., 2011; Kumagai et al., 2013; Atienza et al., 2014) built on the DF- and PVI-based strategies. This study shows that the performance of individual techniques alone is insufficient in identifying the active AF spatial sites (Figures 1, 2). To overcome this, we developed a similarity score that can help identify the active AF spatial sites by a combination of multiple methods.

We developed a similarity score that can bring together various signal processing approaches and successfully identify active AF sites based on the correlation between all the approaches. In this study, we demonstrated the performance of the similarity score based on four approaches, namely, MSF, MSE, DF, and Kt, to identify abnormal electrical sites in patients with unsuccessful AF termination. However, the similarity score might be extended and optimized by incorporating other iEGM analysis approaches and/or by combining a different number of approaches.

In this study, we have used a custom 3D visualization software previously developed (VIEgram) to perform a visual inspection of clinically recorded iEGMs and implement the previously developed novel iEGM analysis techniques (DF, MSF, MSE, and Kt). The recorded sites were distributed in these anatomical regions of the atria, LA appendage, lateral wall, roof wall, posterior wall, inferior wall, septal wall, LSPV, and LIPV. It was observed that the use of these individual approaches fails to robustly identify spatial sites for ablation; the 3D representations of individual methods among the same patients are visually not similar in the distribution of the metrics, thus increasing the difficulty and time consumed to interpret the different approaches at the same time, especially in patients with unsuccessful AF termination. To overcome this, in this study, we have developed a similarity score that combines all the individual approaches to provide the identification of the active AF site.

### Limitations

There are several limitations to our study. First, we have performed only a retrospective analysis of the BiEGMs recorded from a small cohort of patients (*m* = 8) with persistent AF, using a newly developed similarity score. While there is a clear difference in similarity scores between patients with successful and unsuccessful AF ablation, we could only suggest that this method pointed out the active sites of AF. However, to directly demonstrate that these active sites are, in fact, affecting AF ablation outcomes, a prospective study in a cohort of larger patients has to be designed.

Successful AF ablation is defined as the absence of recurrence in AF episodes longer than 30 s in the long-term follow-ups ranging from 3 months to 1 year (Calkins et al., 2007), i.e., the recurrence of the first AF episode, or any atrial tachyarrhythmia post-ablation is perceived as a failure. In this study, the primary procedural endpoint for a successful termination was the termination of AF without electrical cardioversion. The successful termination of AF was defined as the conversion of AF into sinus rhythm by ablation and without electrical cardioversion. The acute success of the procedure was considered as the endpoint in this study. The long-term success of the ablation procedure was not the primary focus of this study but rather the acute success of the ablation procedure in the termination of the AF rhythms.

In this retrospective study, the BiEGMs were only from the LA and were distributed in the following anatomical regions of the atria: LA appendage, lateral wall, roof wall, posterior wall, inferior wall, septal wall, LSPV, and LIPV. However, the exact location of the spatial sites could not be identified due to the limitation of the data. All the approaches used in this study identify the AF activity present on the endocardial surfaces but do not identify intramural abnormal electrical activities. In the future, a prospective clinical study needs to be performed to validate the performance of the proposed similarity score and improve and add more approaches to make it more robust.

In this study, we have not examined the contribution of each feature but assigned equal weights. In the future, we need to determine the contribution of the various methods individually, also add more methods, and then optimize their contributions toward a final similarity score through a weighted sum approach. A larger data will be needed for this approach.

In this study, all the iEGMs were between the durations of 5–15 s. We have shown previously that the individual approaches are robust under reduced time durations (Annoni et al., 2018; Arunachalam et al., 2018), and therefore, we did not expect any significant impact of different time series duration of our results. With respect to spatial samples, the EMD is applied to the measures from the individual approaches at each spatial site and, therefore, will have no significant impact on the scores. In this study, the influence of the duration of the iEGM recordings on the similarity score was not studied.

## Conclusion

In this retrospective study, we investigated the performance of individual BiEGM analysis approaches (DF, MSF, Kt, and MSE) and newly developed similarity scores to identify potential, abnormal, electrically active sites in patients with previously unsuccessful AF termination. The major findings of this study are as follows: (1) individual approaches can discriminate between patients with successful and unsuccessful AF termination but fail to robustly identify spatial sites with active AF drivers, (2) a novel EMD-based similarity score was developed and validated to identify the active AF sites in patients with unsuccessful AF termination, and (3) there was no single common region in the atria associated with active AF sites in patients with unsuccessful AF termination, thus indicating the need for patient-specific mapping and ablation therapy.

## Data Availability Statement

The raw data supporting the conclusions of this article will be made available by the authors, without undue reservation.

## Ethics Statement

The studies involving human participants were reviewed and approved by the University of Minnesota’s Institutional Review Board (IRB: STUDY00003128). The patients provided written informed consent for ablation and data collection for research purposes.

## Author Contributions

EGT, HR, and VR contributed to conceptualization. EGT and VR contributed to methodology. VR, ST, and XK contributed to software and scripts. VR contributed to validation and writing – original draft preparation. VR and ST contributed to formal analysis. VR, ST, XK, HR, and EGT contributed to writing – review and editing. ET contributed to supervision, project administration, and funding acquisition. All authors contributed to the article and approved the submitted version.

## Conflict of Interest

The authors declare that the research was conducted in the absence of any commercial or financial relationships that could be construed as a potential conflict of interest.

## Publisher’s Note

All claims expressed in this article are solely those of the authors and do not necessarily represent those of their affiliated organizations, or those of the publisher, the editors and the reviewers. Any product that may be evaluated in this article, or claim that may be made by its manufacturer, is not guaranteed or endorsed by the publisher.
