# Supplementary material for: Similarity Score for the Identification of Active Sites in Patients With Atrial Fibrillation
Source: Front Physiol. 2022 Jan 20;12:767190. doi: 10.3389/fphys.2021.767190 (PMC8811172; doi:10.3389/fphys.2021.767190)
Supplement: Supplementary file 1 [file Data_Sheet_1.pdf]

Supplemental Material

Analysis Techniques

1. *Patients Baseline Characteristics*

	Successful AF termination	Unsuccessful AF termination	Statistical comparison (p)
Age	61.75 ± 10.81	61.75 ± 13.05	0.9
Sex (M (%))	2 (50)	4 (100)	0.42
LVEF (%)	55 ± 16.83	55 ± 13.54	0.9
LA Size (cm)	5.05 ± 0.48	4.48 ± 0.33	0.04
BMI	29.68 ± 6.77	37.35 ± 8.78	0.34
Hypertension	3 (75)	4 (100)	1
DM	1 (25)	2 (50)	1
OSA	2 (50)	2 (50)	1

Table S.1. Baseline characteristics of persistent AF patients. (LVEF – Left ventricular ejection fraction; LA size – Left atrium size; BMI – Body Mass Index; DM – Diabetes Melitus; OSA – Obstructive Sleep Apnea. All values are expressed as mean±sd or male(%))

2. *Frequency and Temporal Signal Analysis Techniques:*

Information on the novel analysis approaches are listed in Table 1 provided below. The Table is shown here with permission from previous authors [1]. The mathematical equations and descriptions with references are provided. The MSF is frequency-based approach and MSE, SE are entropy based temporal approaches. Kt is a statistical approach that uses the amplitude data of signals.

Method	Equation	Variable Definition	Description	[Ref]
**Multiscale Frequency (MSF)**	$MSF = \rho_0 \left[\sum_{i=1}^{N-1} q_i\right]^{-1} \sum_{i=1}^{N-1} 2^{i+0.5} q_{i+1}$	ρ: *local MSF estimate*<br>q_i: *Output of the ith log-Gabor Filter*<br>ρ_0: *center frequency of the first log-Gabor filter*	*Calculates the instantaneous frequency using the signal spectrum of the voltage intensity time series.*	[2]
**Shannon Entropy (SE)**	$SE = \sum_{i=0}^{N-1} -p_i \log_2 p_i$	*N: Number of amplitude bins*<br>*p: probability of a sample falling within a particular amplitude bin*	*Quantifies uncertainity in voltage intensity distribution.*	[3]

**Kurtosis (Kt)**	$$Kt = E\left\{\left[\frac{x(t) - E[x(t)]}{\sigma}\right]^4\right\}$$	*E: Expected Value*<br>*x(t): Voltage intensity time series*<br>σ: *Variance of the voltage intensity time series*	*Quantifies the 'peakedness' of the voltage intensity signal.*	[5]
**Multiscale Entropy (MSE)**	$$MSE = -\log\left(\frac{A}{B}\right)$$	*A: number of matched vector pairs of length m+1 from the moving average time series*<br>*B: number of matched vector pairs of length m from the moving average time series*	*Calculates the moving average of the voltage intensity time series and calculates the regularity and repetitiveness of the data*	[4]

Table S.2 Summary of Signal Processing Techniques taken with permission from [1]

3. *Similarity Measure Tables for All patients:*

P2

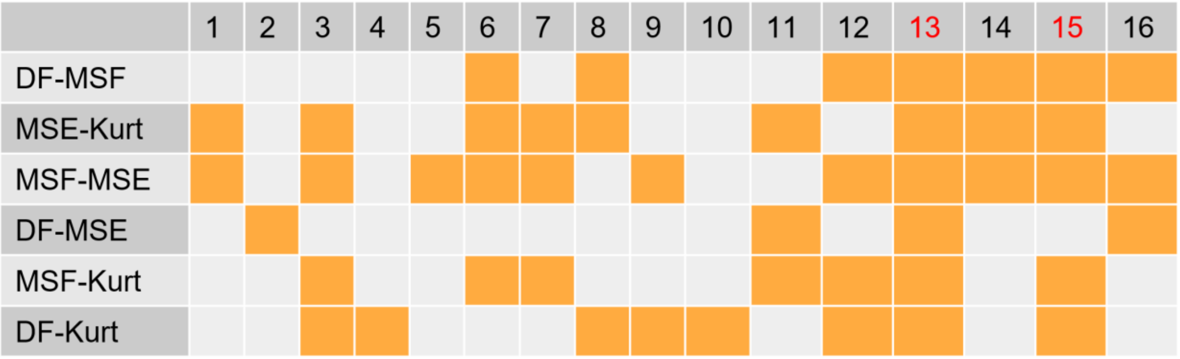

Table S.3. EMD correlation between different pairs of approaches for various spatial sites in patient P2

P3

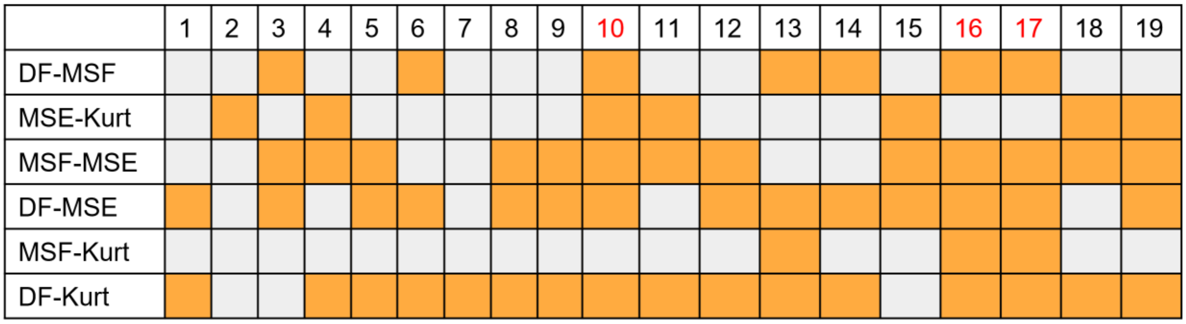

Table S.4. EMD correlation between different pairs of approaches for various spatial sites in patient P3

P4

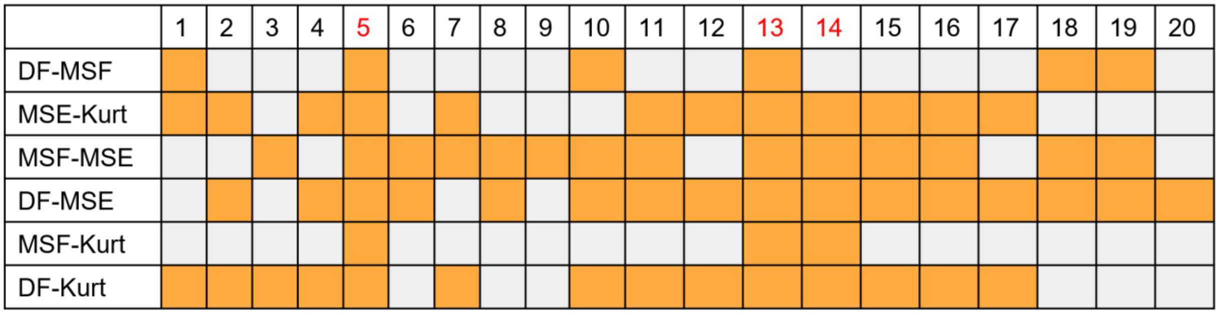

Table S.5. EMD correlation between different pairs of approaches for various spatial sites in patient P4

P5

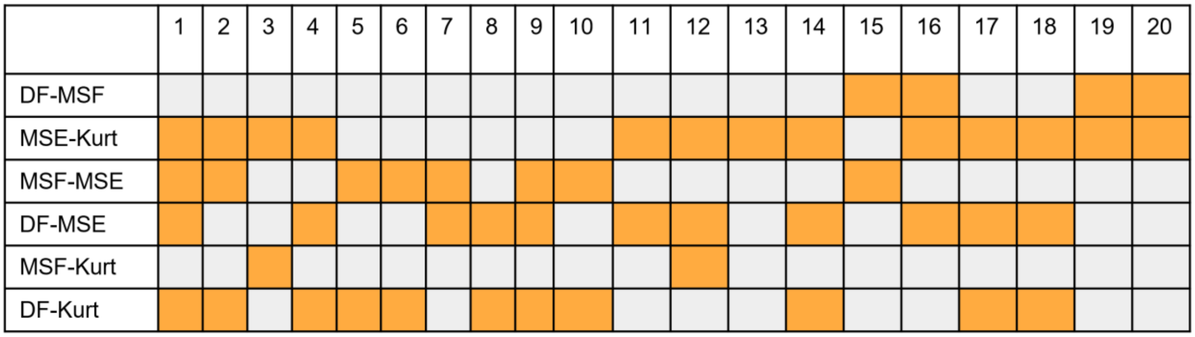

Table S.6. EMD correlation between different pairs of approaches for various spatial sites in patient P5

P6

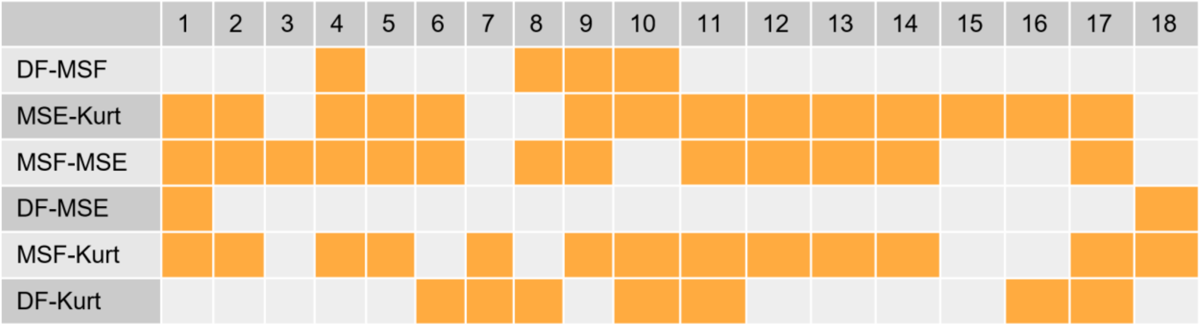

Table S.7. EMD correlation between different pairs of approaches for various spatial sites in patient P6

P7

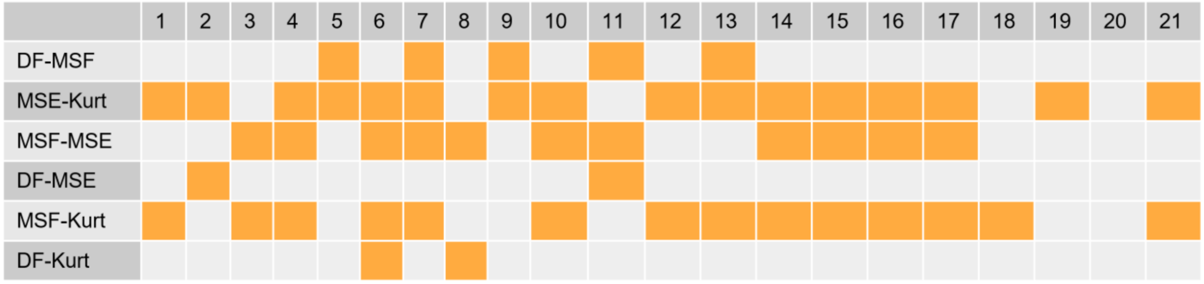

Table S.8. EMD correlation between different pairs of approaches for various spatial sites in patient P7

References:

[1] Annoni EM, Arunachalam SP, Kapa S, Mulpuru SK, Friedman PA, Tolkacheva EG. Novel quantitative analytical approaches for rotor identification and associated implications for mapping. IEEE Transactions on Biomedical Engineering. 2018; 65:273–281.

[2] Arunachalam SP, Annoni EM, Mulpuru SK, Friedman PA, Tolkacheva EG. Novel Multiscale Frequency Approach to Identify the Pivot Point of the Rotor. Journal of Medical Devices, Transactions of the ASME. 2016.

[3] Arunachalam SP, Mulpuru SK, Friedman PA, Tolkacheva EG. Feasibility of visualizing higher regions of Shannon entropy in atrial fibrillation patients. Proceedings of the Annual International Conference of the IEEE Engineering in Medicine and Biology Society, EMBS. 2015:4499–4502.

[4] Arunachalam SP, Kapa S, Mulpuru SK, Friedman PA, Tolkacheva EG. Improved Multiscale Entropy Technique with Nearest-Neighbor Moving-Average Kernel for Nonlinear and Nonstationary Short-Time Biomedical Signal Analysis. Journal of Healthcare Engineering. 2018.

[5] Arunachalam SP, Annoni EM, Mulpuru SK, Friedman PA, Tolkacheva EG. Kurtosis as a statistical approach to identify the pivot point of the rotor. Annual International Conference of the IEEE Engineering in Medicine and Biology Society. IEEE Engineering in Medicine and Biology Society. 2016; 497-500.
